# Seedling resistance and chemical defenses against *Psylliodes chrysocephala* : the roles of seed age and sinapinic acid in *Sinapis alba* and *Brassica napus*

**DOI:** 10.1002/ps.70724

**Published:** 2026-03-18

**Authors:** Daniel Rüde, Samantha M Cook, Bernd Ulber, Tobias Pöhnl, Michael Rostás

**Affiliations:** ^1^ Division of Agricultural Entomology Georg‐August University Göttingen Germany; ^2^ Rothamsted Research Harpenden UK; ^3^ Division of Quality and Sensory of Plant Products Georg‐August University Göttingen Germany

**Keywords:** cabbage stem flea beetle, feeding preference, host plant resistance, oilseed rape, rapeseed, white mustard

## Abstract

**BACKGROUND:**

The cabbage stem flea beetle, *Psylliodes chrysocephala*, is a major pest of winter oilseed rape (*Brassica napus*) in Europe. Adults feed on cotyledons and young leaves, threatening the establishment and vigor of the crop. With no insect‐resistant cultivars of *B. napus* currently available, farmers must rely primarily on synthetic insecticides for pest control. This study investigated the resistance of *Brassica napus* and *Sinapis alba* seedlings to adult *P. chrysocephala* feeding and explored the underlying chemical defense mechanisms. Seedlings from three *B. napus* accessions, including two with contrasting seed ages, and three *S. alba* accessions were evaluated for feeding damage in controlled laboratory conditions, while a subset of two *B. napus* and two *S. alba* accessions was further evaluated in semi‐field assays. Central metabolomic and glucosinolate profiling was conducted to identify resistance‐linked compounds.

**RESULTS:**

Under controlled conditions, *S. alba* seedlings exhibited substantially less feeding damage compared to a standard *B. napus* cultivar under controlled conditions. Notably, older *B. napus* seeds stored for 6 or 9 years produced seedlings with significantly reduced susceptibility to herbivory, coinciding with changes in central and specialized metabolite profiles, compared to freshly harvested seeds (younger than one year). Glucosinolate analyses revealed species‐specific profiles, while dual‐choice bioassays identified sinapinic acid as a feeding deterrent to *P. chrysocephala* adults.

**CONCLUSION:**

We successfully identified variation in cotyledon‐stage resistance to *P. chrysocephala* among Brassicaceae species. These findings highlight the potential of *S. alba* as a source of resistance traits and suggest that seed physiology influences seedling resistance, offering new avenues for breeding insect‐resistant oilseed crops, which are needed for integrated pest management. © 2026 The Author(s). *Pest Management Science* published by John Wiley & Sons Ltd on behalf of Society of Chemical Industry.

## Graphical abstract

The cabbage stem flea beetle (*Psylliodes chrysocephala*) is a major pest of winter oilseed rape (*Brassica napus*). We tested *B. napus* and *Sinapis alba* seedlings for feeding resistance and underlying chemical defenses. *S. alba* was least damaged, while metabolite profiles revealed resistance‐linked compounds, highlighting breeding potential for insect‐resistant oilseed crops.

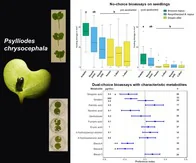

## 1 INTRODUCTION

Oilseed rape (*Brassica napus*) is an important agricultural crop, significantly contributing to global vegetable oil production and serving as livestock feed. It is the most important oil crop in the European Union.^1^ However, *B. napus* is susceptible to various insect pests throughout the growing season, which can pose substantial threats to yield.^2^ Among these pests, the cabbage stem flea beetle (*Psylliodes chrysocephala*; Coleoptera: Chrysomelidae) is currently regarded as the most destructive in Central Europe.^3^, ^4^

*Psylliodes chrysocephala* adults undergo a summer diapause, known as aestivation.^5^, ^6^ Following this period, post‐aestivation adults migrate to newly emerging *B. napus* crops, where they feed on cotyledons and early leaves.^7^ Although *B. napus* can compensate for moderate feeding damage during early growth stages,^8^ severe infestations can threaten crop establishment, as demonstrated in England in 2014, when almost 100% of crops were lost in the East of England region.^9^

The larvae of *P. chrysocephala* mine within petioles and stems during autumn and winter, increasing the crop's susceptibility to frost damage.^10^, ^11^ Larvae drop from the plant to pupate in the soil with new generation (pre‐aestivation) beetles emerging in late spring/ early summer. Since the ban on neonicotinoids, pyrethroids have become the only effective direct control measure available.^3^ This challenge is further exacerbated by the growing resistance of *P. chrysocephala* populations to pyrethroids.^12^, ^13^

The escalating threat posed by *P. chrysocephala* highlights a critical gap in the integrated pest management of *B. napus*: the lack of insect pest‐resistant cultivars.^14^ Plant resistance to insect pests can be conferred through a variety of mechanisms, including morphological traits (e.g., trichomes), physical barriers (e.g., wax layers), and biochemical traits such as the production of antibiotic metabolites which can reduce insect performance.^15^

A thorough evaluation of potential resistance mechanisms requires a comprehensive understanding of plant–herbivore interactions. Among these mechanisms, biochemical resistance is especially promising, as it can influence nearly every stage of the plant–herbivore relationship.^16^ For example, the host‐plant location of herbivores can be disrupted by altering the composition of emitted plant volatiles, while host acceptance for feeding or oviposition can be prevented by modifying surface metabolites on leaves.^17^ Another effective strategy, as suggested by Hervé et al. (2014),^18^ is to reduce herbivore feeding by changing the chemical composition of specific plant tissues. Since insect gustatory neurons can be stimulated by either phagodeterrent or phagostimulant compounds,^19^ manipulating the metabolite profile within plant tissues can decrease feeding stimulation or enhance deterrence, thereby conferring resistance.^18^

When identifying target compounds for manipulating plant–herbivore interactions, it is crucial to consider both central and specialized metabolites, as well as their interactions, since both classes can significantly influence insect feeding behavior.^18^ Central metabolites, such as sugars, amino acids, and organic acids, are essential for plant growth and development and are typically constitutively present in plant tissues.^20^ In contrast, specialized metabolites — including glucosinolates (GLS), alkaloids, and phenolics — often fulfill functions in plant defense and can be upregulated in response to herbivory.^21^, ^22^, ^23^

Several studies have explored the role of glucosinolates in the feeding behavior of flea beetles (*Phyllotreta* spp. and *P. chrysocephala*), as these compounds are characteristic for Brassicaceae and serve as defense against generalist herbivores but act as kairomones for specialist herbivores.^24^ However, research on GLS in relation to *P. chrysocephala* has produced inconsistent results.^25^ By investigating the influence of GLS composition in *B. napus* seedlings on *P. chrysocephala* feeding, it was found that GLS levels did not significantly affect feeding activity.^26^ Notably, adult *P. chrysocephala* were also stimulated to feed on sucrose and other sugars incorporated into agar, suggesting that GLS may not be the only determinants of feeding preferences.^26^ In contrast, Williams (1989)^27^ reported that a *B. napus* cultivar with very low seed GLS content suffered the most damage at the cotyledon stage. Furthermore, a positive correlation between leaf GLS levels and leaf damage was observed, underscoring the complex and context‐dependent relationship between plant chemistry and *P. chrysocephala* feeding behavior.^28^

Given the limited resistance detected within *B. napus* itself, other Brassicas such as *Sinapis alba* have been included in screening programs as potential sources of resistance.^14^, ^18^ In Canada, researchers have consistently demonstrated that *S. alba* seedlings exhibit resistance to flea beetle (*Phyllotreta* spp.) feeding.^29^, ^30^, ^31^ Mechanically wounding a cotyledon of *S. alba* induced increased resistance to *Phyllotreta* in both the undamaged cotyledon and the first true leaf—a response not observed in *B. napus* and *B. rapa*.^32^ Additionally, *S. alba* is better able to compensate for cotyledon damage, whether caused artificially or by *Phyllotreta* species, making it a promising candidate for breeding tolerant cultivars.^33^, ^34^ Furthermore, interspecific hybrids of *S. alba × B. napus* displayed seedling resistance to *Phyllotreta* feeding,^30^ highlighting the potential for transferring resistance traits from *S. alba* to *B. napus*. While these studies focused on *Phyllotreta* species, Döring & Ulber (2020)^35^ observed lower larval weight and higher larval mortality of *P. chrysocephala* in *S. alba* (cv. Accent) than in *B. napus* (cv. Robust) plants. However, in dual‐choice tests, adult *P. chrysocephala* showed no clear feeding preference between the true leaves of *S. alba* and *B. napus*.^36^

This study focused on screening various *Brassica* accessions at the cotyledon growth stage for resistance against feeding of adult *P. chrysocephala*. Given that *B. napus* plants are particularly vulnerable to herbivore attacks at this early growth stage, the primary goal was to identify genotypes and metabolites that could confer reduced susceptibility to this pest, thereby informing future breeding strategies.

Our study involved no‐choice feeding assays using *B. napus* and *S. alba* lines, with both pre‐ and post‐aestivated beetles, to assess if beetle physiological state influences feeding behavior. Comprehensive metabolomic analyses were performed on the test seedlings of different seed ages, focusing on primary metabolites and glucosinolate profiles. Candidate compounds emerging from these analyses were subsequently evaluated in dual‐choice leaf disc assays to determine their role in *P. chrysocephala* feeding. This approach directly addresses the critical gap in insect resistance in *B. napus* by exploring potential resistance traits and supporting the development of *B. napus* cultivars with enhanced resistance to *P. chrysocephala*.

## 2 MATERIALS AND METHODS

### 2.1 Plants

Six different genotypes were tested in our experiments with some variation in seed age. The test accessions ‘Resynth’ (*B. napus*), ‘S.alba1’ and ‘S.alba2’ (both *S. alba*) were provided by the owner breeding companies KWS SAAT SE & Co. KGaA (Einbeck, Germany) and NPZ Innovation GmbH (Holtsee, Germany). Winter oilseed rape, *B. napus* cv Arabella (Limagrain GmbH, Edemissen, Germany) and white mustard, *S. alba* cv. Passion (Deutsche Saatveredelung AG, Lippstadt, Germany) were included as standards in the experiments. Both cultivars are commonly cultivated in Germany, as an oilseed crop and a cover crop, respectively. The *B. napus* accession ‘Resynth’ is a resynthesized line, meaning that the genesis of *B. napus* was artificially recreated by hybridizing the two progenitor species, *Brassica rapa* and *Brassica oleracea*. Preliminary trials including ‘Resynth’ seed lots with different seed ages indicated altered susceptibility towards adult *P. chrysocephala* feeding, so we took the opportunity to further investigate possible effects of seed age and underlying mechanisms of the observed variation. Seedlings grown from newly propagated seeds (seed age ~ 1 year) are referred to as ‘Resynth (new)’ and seedlings grown from seeds stored for 9 years are designated as ‘Resynth (old)’. The genetic homogeneity of both seed batches of ‘Resynth’ was confirmed by NPZ Innovation GmbH using 20 SNP markers distributed across all 19 chromosomes of the *Brassica napus* genome and detected via KASP marker technology. For each original seed lot, at least 23 individual plants were analyzed. The effect of seed age was further investigated by comparing the susceptibility of *B. napus* seedlings cv. Adelmo (KWS SAAT SE & Co. KGaA) grown from seeds stored for six years (harvested in 2016) with those grown from freshly harvested seeds obtained four months prior to the experiment.

### 2.2 Insects

#### 2.2.1 Pre‐aestivated beetles

*Brassica napus* plants cv. Bender (Deutsche Saatveredelung AG, Lippstadt, Germany) infested by *P. chrysocephala* were collected in January 2022 from an insecticide‐free crop (cv. Bender) near Göttingen. Larvae were extracted live by dessiccating the plants on a wire grid suspended inside a plastic box (*sensu* Seimandi‐Corda et al. 2024^37^). Larvae dropping into the box were identified as *P. chrysocephala* by their characteristic thoracic legs, black prothoracic plate and anal plate with spines.^2^, ^7^ Third instar larvae^7^ were regulary collected over a 2 ‐ 3 week desiccation period and transferred to plastic containers filled with a 1:1:1 (v/v) mixture of moist sand (0.1 mm–2 mm diameter; Oppermann Kiesgewinnungs‐ und Vertriebs‐GmbH, Hannoversch Münden, Germany), loam (obtained from field subsoil in Pöhlde, Germany), and potting soil (Fruhstorfer Erde Type P 25, HAWITA GmbH, Vechta, Germany) for pupation. Containers were stored at 5°C to decelerate insect development. Prior to experiments, the emergence of adult beetles was induced by moving the containers to a controlled environment room (20.2°C ±1, 60.4%±6 RH, 16 h:8 h L:D). Adults were collected daily and used in experiments within 1‐3 days after emergence. The adult beetles were maintained in mesh cages (BugDorm‐4M2222, Megaview Science Co., Ltd., Taichung, Taiwan) and were provided with fresh *B. napus* leaves (cv. Arabella) *ad libitum* as a food source. Beetles were starved for 24 hours with access to water before being used in experiments to standardize their feeding motivation.^26^

#### 2.2.2 Post‐aestivated beetles

*Psylliodes chrysocephala* adults were collected from an insecticide‐free crop near Göttingen following their aestivation in October 2022, belonging to the same generation as the pre‐aestivated beetles. Collection took place in a young crop (BBCH 12‐14; cv. Bender), using a sweep net and a fuel‐powered vacuum sampler (SH56, Stihl AG & Co.KG, Waiblingen, Germany). The beetles were kept in mesh cages (BugDorm‐2120, MegaView Science Co.) under controlled conditions (16°C, 16 h:8 h L:D) with a constant supply of *B. napus* plants (cv. Arabella). Before experimental use, beetles were starved for 24h with access to water.

Prior to the experiments, the sex of all pre‐and post‐aestivated beetles was determined based on the differing size and shape of the tarsal segments of their front legs ensuring an even distribution of females and males in each experiment.^38^

### 2.3 No‐choice feeding bioassays

Seedlings for no‐choice bioassays were cultivated by sowing individual seeds into plastic tubes (50 mL Falcon, Corning, USA) provided with a drainage hole at the conical base. Each tube was filled with a 1:1 (v/v) mixture of sand (0.1 mm–2 mm diameter; Oppermann Kiesgewinnungs‐ und Vertriebs‐GmbH) and potting soil (Fruhstorfer Erde Type P 25, HAWITA GmbH, Vechta, Germany). Plants were grown in a climate‐controlled chamber maintained at 20.2 ± 1 °C and 60.4 ± 6% RH, under illumination from high‐pressure sodium lamps (HS.TP400, Hortilux Schréder, Monster, Netherlands) with a 16 h:8 h light:dark photoperiod. Seedlings were used for bioassays once their cotyledons were fully expanded (BBCH growth stage 10; Lancashire *et al*. 1992^39^). All seeds, unless otherwise specified, were approximately one year old at the time of sowing.

In each no‐choice bioassay, the tube with a test seedling was inserted into the center of a plastic foam disc (12 cm diameter) (Supporting Information, Fig S1), with the rim of the tube flush with the surface of the disc. This assembly was then placed inside a clear plastic cup (0.33 L; 12 cm diameter), ensuring that the foam disc fitted tightly against the cup walls to prevent beetles from accessing the area beneath the disc (Supporting Information, Fig. S1). A single beetle was introduced into each cup, which was then sealed with a domed plastic lid perforated for ventilation. The bioassays were conducted under the same controlled environmental conditions used for seedling cultivation. After 48 h, the cotyledons were excised, and the area consumed was digitally quantified as described below (section 2.4).

In separate no‐choice bioassays, pre‐aestivated and post‐aestivated adults were tested. The accessions evaluated in both experiments were *B. napus* cv. Arabella, ‘Resynth’ (old), ‘Resynth’ (new), and *S. alba* cv. Passion, ‘S. alba1’, and ‘S. alba2’. The experiment using pre‐aestivated beetles was conducted over three days in April‐May 2022 with a total sample size of n = 14. Post‐aestivated beetles were tested over two days in October 2022 (n = 18). In a third no‐choice bioassay, pre‐aestivated beetles were tested over two days in August‐September 2022 to compare the susceptibility of old versus new seeds of the *B. napus* cultivar ‘Adelmo’ (n = 16).

### 2.4 Damage assessment

To assess the consumed leaf area in each feeding experiment, excised cotyledons were placed on a neutral white (4000K) LED light panel (CLPGA40/M/54, Tegral Lighting, Bishop's Stortford, England) and photographed. The images were assessed by measuring the consumed leaf area using an imaging software (ImageJ version 1.53k, U.S. National Institutes of Health, Bethesda USA).

### 2.5 Semi‐field experiment

Multipot seedling trays (31 × 53 × 5.5 cm; HerkuPlast‐Kubern GmbH, Ering am Inn, Germany) containing 6 × 10 cells (4.6 cm diameter per cell) were used for seedling cultivation. Each cell was filled with a 1:1 (v/v) mixture of sand and potting soil, as previously described. In both the second and fifth rows of each tray, one seed was sown in every alternate cell, resulting in a total of 10 plants per tray. Each tray was planted exclusively with a single accession. Seedlings were grown in an unheated greenhouse under ambient environmental conditions until they reached the fully expanded cotyledon stage (BBCH 10).

The semi‐field experiment was conducted in a newly emerging crop of *B. napus* cv. Bender in a commercial crop near Göttingen (coordinates: 51°33'56.4" N 9°56'45.8" E). At the start of the trial, on 12 September 2022, the crop was at BBCH stages 10‐12. Five accessions (‘Arabella’, ‘Resynth (old)’, ‘Resynth (new)’, ‘Passion’ and ‘S.alba2’) were tested with eight replicates. Accession ‘S.alba1’ was excluded from the semi‐field experiment because of resource limitation. The multipot‐trays were arranged in a randomized complete block design with eight blocks (total: 40 multi‐pot trays). The trays were placed directly on the soil surface within the crop, with a spacing of 1 m between trays within each row and 2 m between blocks. After a four‐day exposure period, the trays were transported to the laboratory for digital assessment of feeding damage, as described in Section 2.4.

### 2.6 Metabolome analyses

The seedlings for all metabolite analyses were cultivated as described for the no‐choice bioassays.

#### 2.6.1 Central metabolome

To prepare samples for analysis, cotyledons were cut, lyophilized, and pooled to yield 40 mg per replicate, with 6 replicates per accession. The pooled samples were ground into a fine powder and suspended in 120 μL methanol (99.95% v/v GC‐MS grade). As an internal standard, 20 μL of ribitol (20 ng μL^−1^ in 99.95 % LC‐MS grade methanol) was added prior to solvent removal using a rotary vacuum evaporator (RVC 2‐25 CD plus, Christ, Osterode am Harz, Germany) at 30 °C for 180 min. The samples were stored overnight at −80 °C under argon to prevent oxidation. The following day, 80 μL of methoxyamination reagent (20 mg mL^−1^ methoxyamine hydrochloride in pyridine) was added to each sample. After centrifugation for 1 min, samples were placed on a shaker for 90 minutes. For analysis, 20 μL of the derivatized sample was transferred into a GC glass vial and 20 μL N‐methyl‐N‐(trimethylsilyl) trifluoracetamide (MSTFA) was added for derivatization and vortexed for 10 seconds.

The samples were analyzed using a gas chromatograph‐mass spectrometer (GC‐MS) system (GC 7890B coupled with MS 5977B, Agilent Technologies). The GC was equipped with a Restek Rtx‐5 w/Integra‐Guard column (30 m × 0.25 mm ID × 0.25 μM df), an inlet liner (split liner 5183‐4647, Agilent Technologies), and operated in split mode with a split ratio of 20:1. A PAL autosampler (PAL RSI 85, Switzerland) was used for injection of 1 μL per sample at an injection temperature of 250 °C. Helium at a flow rate of 1.2 mL min^−1^ was used as carrier gas. The initial oven temperature was set at 70 °C held for 2 min, then gradually increased to 325 °C with a ramp rate of 5 °C min^−1^ and maintained for 10 min. The instruments were operated with the software MassHunter Workstation GC/MS Acquisition (ver. 10.0.368, Agilent Technologies), using a scan range of 70 m/z to 650 m/z and a solvent delay of 9.9 min.

For tentative identification of the derivatized metabolites, the retention indices and mass spectra were compared to the Golm Metabolome Database (GMD), BinBase database, Wiley 11 and NIST 17 using the softwares MS‐DIAL (ver. 4.8) and MSD ChemStation (ver. F.01.03.2357, Agilent). For confirmation, commercial authentic standards of 4‐hydroxybenzyl alcohol, D‐gentiobiose, palmitic acid (Carl Roth GmbH, Karlsruhe, Germany), sinapinic acid, fumaric acid (Merck KGaA, Darmstadt, Germany), 4‐hydroxybenzoic acid, erucic acid and nicotinic acid (Fisher Scientific GmbH, Schwerte, Germany) were analyzed and compared with the GC‐MS results. Quantification was achieved by comparing peak areas of the respective metabolite and the ribitol standard.

#### 2.6.2 Glucosinolates (GLS)

Cotyledons were cut and immediately frozen in liquid nitrogen. The extraction and analysis of desulfo‐GLS was conducted as described by Bayer *et al*. (2022),^40^ with 5 replicates per accession. Briefly, GLS were extracted three times from 20 mg lyophilized and grounded sample material using 70% hot methanol (750 μL initially, followed by 2 × 500 μL at 70 °C). Sinigrin (100 μM) was added as internal standard. The supernatants were combined and loaded onto a column (0.5 mL bed volume) containing acetic acid‐activated DEAE Sephadex A‐25 resin (Cytiva, Marlborough, MA, USA). Desulfatation was performed overnight at room temperature by adding 75 μL of β‐glucuronidase/arylsulfatase enzyme solution. The desulfo‐GLS were eluted by applying 0.5 mL of ultrapure water twice, and the eluates were subsequently filtered through Spin‐X cellulose filters (0.22 μM; Corning Costar Spin‐X, Sigma Aldrich, St. Louis, MI, USA) by centrifugation at 3000 rpm.

The GLS were separated using high performance liquid chromatography (HPLC) (Jasco 4000 series, JASCO Corporation, Tokyo, Japan), equipped with a PU‐4185 pump, AS‐4150 autosampler and a UV‐4070 UV/vis detector. The operating software was ChromNav (Vers. 2.01.01, JASCO Corporation). A NUCLEODUR Sphinx RP column (150 × 4.6 i.d.; particle size 5 μm, Macherey‐Nagel, Düren, Germany) was used with a NUCLEODUR Universal RP guard column (4 × 3 mm i.d.; particle size 5 μm, Macherey‐Nagel) at a flow rate of 0.6 mL min^−1^. Eluents were ultrapure water (eluent A) and acetonitrile (eluent B) (Carl Roth, Karlsruhe, Germany). The gradient (eluent B) was 1‐20% (1‐20 min), isocratic 20% (20‐25 min), 20‐1% (25‐27 min), and isocratic 1% (27‐35 min) for reequilibration. The injection volume was 20 μL. Quantification was achieved by using peak areas of the internal standard together with reference factors as suggested by Clarke (2010).^41^ Preliminary identification was based on comparison of retention times to those of authentic standards of sinigrin, progoitrin, epiprogoitrin, glucoraphanin, gluconapin, glucobrassicanapin, glucobrassicin and gluconasturtiin (PhytoLab, Vestenbergsgreuth, Germany). To verify the identity of the GLS, mass spectroscopy spectra were analyzed using a LC/Q‐TOF MS (Agilent 6545 quadrupole time‐of‐flight), coupled with an UHPLC system (Agilent 1290 Infinity II) and compared with the literature. Separation for MS analysis was conducted under the same above‐mentioned conditions under a reduced injection volume of 5 μL. GLS were analyzed in positive mode in a range from *m/z* 100 to 920 with a rate of 4 spectra/second. Throughout the analysis the gas temperature was 320 °C at a flow rate of 8 L min^−1^. Nebulizer gas was 35 psi, sheath gas temperature was 350 °C, and sheath gas flow rate was 11 L min^−1^. Source parameters were a capillary voltage of 3500 V, a nozzle voltage of 1,000 V, and a fragmentor voltage of 150 V.

### 2.7 Dual‐choice feeding bioassay using leaf discs

In order to test stimulatory or deterrent effects of selected compounds identified through the metabolome analyses, dual‐choice feeding bioassays with leaf discs were conducted. Nine compounds were selected based on their contrasting abundance among the analyzed accessions: 4‐hydroxybenzyl alcohol, 4‐hydroxybenzoic acid, erucic acid, fumaric acid, gentiobiose, nicotinic acid, palmitic acid, sinalbin and sinapinic acid. Authentic standards, identical to those used in the metabolomic analyses, were used. Sinapinic acid and nicotinic acid were dissolved in acetone, while all other compounds were dissolved in methanol. Each compound was tested at a concentration representative of its naturally occurring level per leaf area. For sinalbin, an additional treatment at a 1000‐fold lower concentration was included to assess potential effects at minimal levels.

In addition to individual compounds, three blends reflecting the characteristic metabolite profile of *S. alba* cotyledons were tested. Blend A consisted of 4‐hydroxybenzoic acid, 4‐hydroxybenzyl alcohol, sinapinic acid, gentiobiose, and palmitic acid, each at their physiological concentrations. Blend B was identical to Blend A, with the addition of sinalbin at its physiological concentration (500 μg/leaf disc). Blend C matched Blend A but included sinalbin at a reduced concentration (0.5 μg/leaf disc).

Leaf discs were taken from *B. napus* plants (cv. Arabella), which had been cultivated in pots (10 × 12 cm) filled with a 1:2 (v/v) mixture of sand and potting soil. Plants were grown in a greenhouse under temperatures ranging from 16 to 23 °C. Supplemental lighting (Elektrox SUPER BLOOM HPS 400 Watt, Grow In AG, Berlin, Germany) was provided at an intensity of 85 μmol m^−2^ s^−1^ to maintain a 16 h:8 h light:dark photoperiod.

The dual‐choice bioassay was prepared by cutting two leaf discs (15 mm in diameter) from either side of the midvein of the third true leaf (BBCH 14) to ensure consistency. For each assay, 20 μL of solvent with the respective test compound was pipetted on the abaxial side of one of the discs (treatment) while 20 μL of the respective solvent was pipetted on the other discs (control). After the solvent had evaporated at room temperature, both discs were placed abaxial side up in a Petri dish (35 × 10 mm, Sarstedt AG & Co. KG, Nümbrecht, Germany) containing 2 mL of 1% water agar (1% Agar‐Agar Kobe 1, Carl Roth). One pre‐aestivated adult *P. chrysocephala* was released into each Petri dish and then placed in a climate chamber (20 °C, ~60%RH and 16:8 L:D). After 48 hours, the leaf discs were photographed for digital quantification of the consumed leaf area as described in Section 2.4. The feeding preference index was calculated to normalize between subsequent bioassays (index = (damage on test disc – damage on control disc)/(damage on test disc + damage on control disc)) according to Austel *et al*. (2021).^42^ Due to limited daily availability of beetles, the compounds were tested on different days, with each compound tested on two or three days to avoid day‐dependent effects. Petri dishes in which no feeding occurred after 48h were excluded from further analyses. The final number of replicates ranged from 16 to 32. Individual beetles were only used once in experiments.

### 2.8 Data analysis

All statistical data analyses and data visualizations were performed with the software ‘R’ v. 4.2.2 and ‘R Studio’ v. 2021.09. The no‐choice feeding bioassays were statistically analyzed using a Generalized Linear Model (Gaussian distribution), since the assumptions of a linear regression were not met. Feeding damage on the accessions in the semi‐field experiment were compared by ANOVA; because of the high number of undamaged seedlings, the mean leaf area consumed per tray was used for analysis rather than a mean per plant. The frequency of damaged plants in the semi‐field trial was compared by using a Generalized Linear Mixed Model with a binomial distribution and ‘block’ as random effect. In both the no‐choice bioassay and semi‐field trial, pairwise post‐hoc tests between accessions were done using Estimated Marginal Means with Tukey adjusted p‐values. The comparison of feeding damage between the two seed age batches of *B. napus* cultivar ‘Adelmo’ was achieved with a t‐test, since all conditions were met. Quantitative comparison of metabolites was done by a Kruskal‐Wallis test and a Pairwise Wilcoxon Rank Sum test with p‐value adjustment according to Benjamini & Hochberg (1995)^43^ and the heatmap was created using the package ‘heatmaply’.^44^ The feeding indices of the dual‐choice assays were statistically compared by a two‐sided Wilcoxon signed rank test to zero with continuity correction.

## 3 RESULTS

### 3.1 No‐choice feeding bioassays

In no‐choice feeding bioassays, significant differences between the accessions were found in regard to the consumed leaf area by pre‐aestivated (χ^2^ = 5585.7, df = 5, *P* < 0.001) and post‐aestivated (χ^2^ = 904.1, df = 5, *P* < 0.001) beetles. All tested *S. alba* accessions and seedlings grown from ‘Resynth (old)’ had significantly less feeding damage than the standard *B. napus* ‘Arabella’ (Figure 1(A), (B)). However, the feeding damage on ‘Resynth (new)’ seedlings was not significantly different from damage on ‘Arabella’ (t = 2.08, df = 77, *P* = 0.309). The pairwise comparisons of the accessions yielded the same pattern of significant differences for the pre‐ and post‐aestivation beetles (Figure 1(A), (B)). Across all accessions, feeding damage was higher when pre‐aestivated beetles were used compared to post‐aestivated beetles. Overall, pre‐aestivated beetles consumed a mean leaf area of 20.8 mm^2^ (SE = 1.8), whereas post‐aestivated beetles consumed only 3.6 mm^2^ (SE = 0.5).

**Figure 1:**
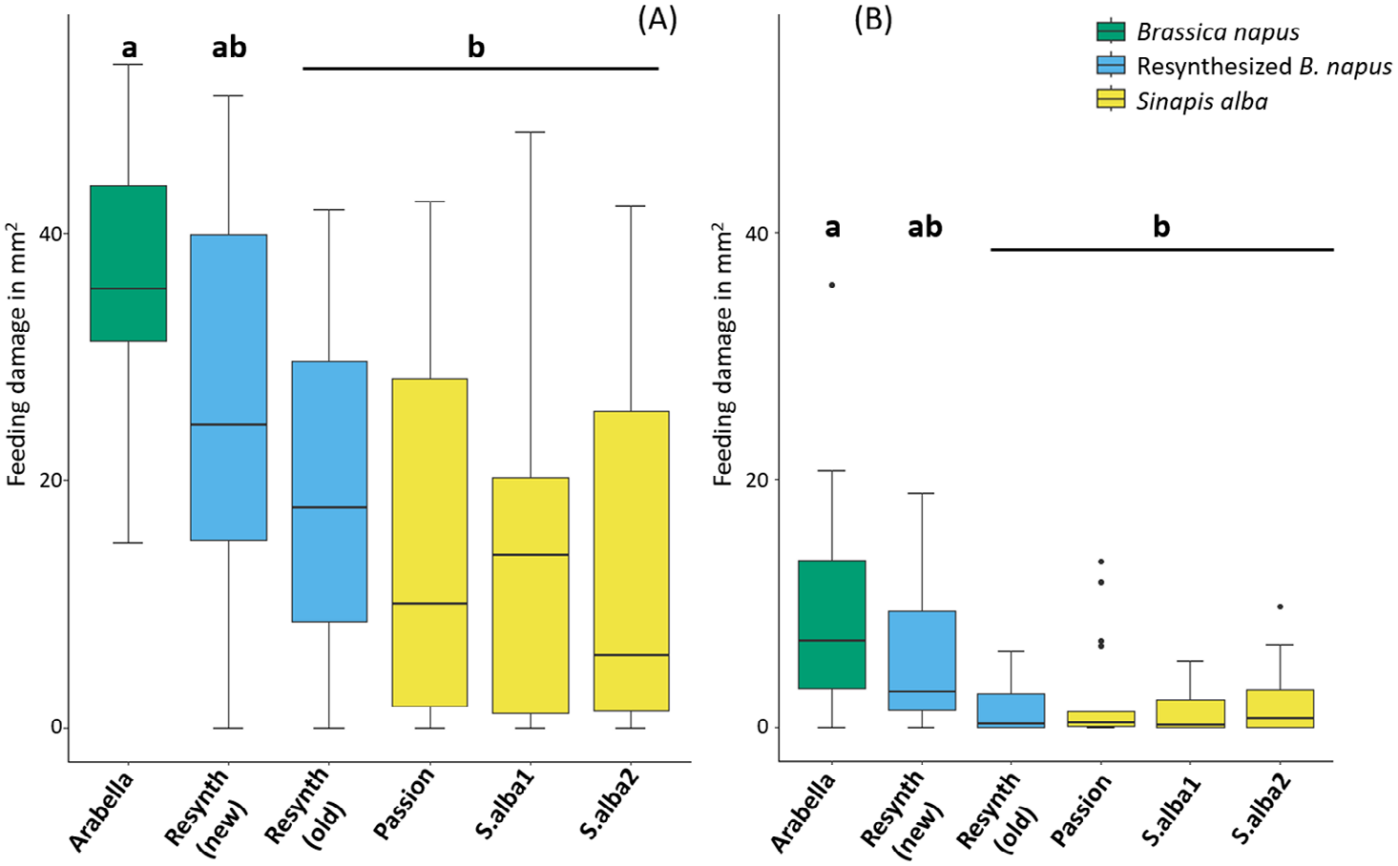
Feeding damage (mm^2^) on seedlings caused by pre‐aestivation (A) and post‐aestivation (B) adult *Psylliodes chrysocephala* in no‐choice bioassays. For each replicate (A: n = 14; B: n = 18), one seedling (BBCH 10) was inserted into a plastic cup and one beetle was allowed to feed for 48 h. Different lowercase letters indicate significant difference (*P* < 0.05) between treatments: *Brassica napus* cv. Arabella, ‘Resynth’ = *B. napus* resynthesized by hybridizing *Brassica rapa* x *Brassica oleracea* using seeds with different seed age (old = 9 years, new = 1 year), *Sinapis alba* cv. Passion and breeders lines ‘S. alba1’ and ‘S.alba2’. The median is shown by the central line in each boxplot, with the lower and upper box edges denoting the 25th (Q1) and 75th (Q3) percentiles.

A similar effect of seed age on seedling acceptance was found with *B. napus* cv. Adelmo. Seedlings grown from 6‐year‐old seeds suffered significantly lower feeding damage than seedlings grown from seeds harvested 4 months prior to the experiment (t = ‐3.08, df = 30, *P* = 0.004; Fig. 2).

**Figure 2:**
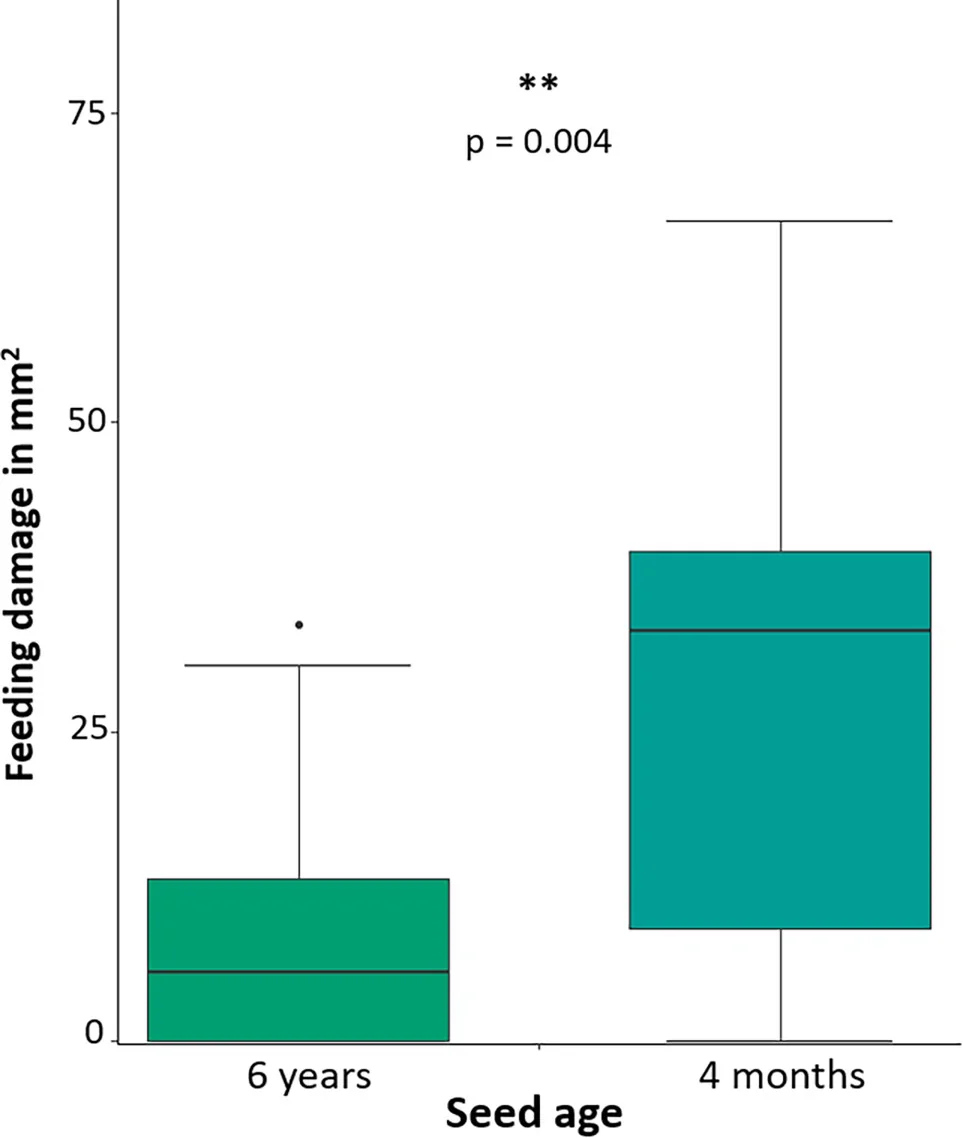
Feeding damage (mm^2^) caused by adult *Psylliodes chrysocephala* in no‐choice bioassays using *B. napus* cv. Adelmo seedlings (BBCH 10) grown from seeds of different ages. Each replicate (n = 16 per seed age) consisted of a single seedling placed in a plastic cup exposed to one beetle (pre‐aestivation) for 48 h. The median is shown by the central line in each boxplot, with the lower and upper box edges denoting the 25th (Q1) and 75th (Q3) percentiles.

### 3.2 Semi‐field experiment

In the field, the tested accessions showed significant variation in susceptibility, as measured by both the proportion of damaged plants and the cotyledon area consumed (F_4,35_ = 3.99, *P* = 0.009) by *P. chrysocephala* adults (Figure 3). The ‘Resynth (old)’ plants suffered a significantly lower proportion of damaged plants (OR = 3.05, 95% CI: 1.12‐8.26, *P* = 0.02) and reduced cotyledon area damaged (t = ‐3.7, df = 35, *P* = 0.006) compared to cv. Arabella. Furthermore, Resynth (old)’ had a significantly lower proportion of damaged plants compared to ‘Resynth (new)’ (OR = 3.21, 95% CI: 1.18‐8.68, *P* = 0.012), although the consumed cotyledon area did not differ significantly (t = 2.52, df = 35, *P* = 0.109) between these two accessions. All other pairwise comparisons were not significantly different.

**Figure 3:**
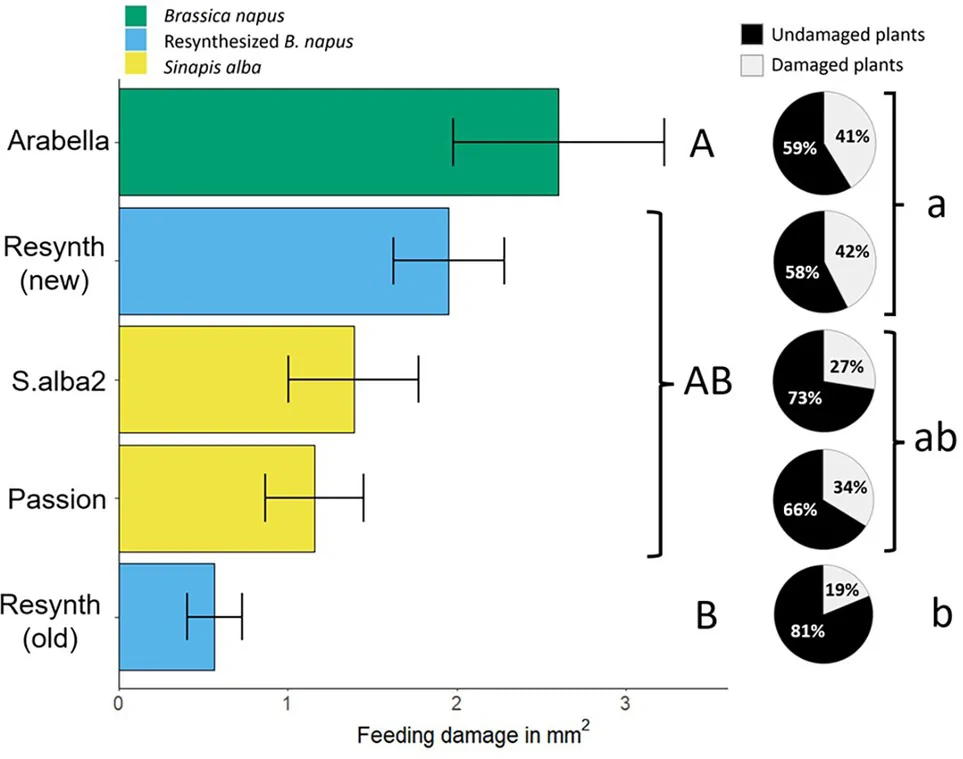
Mean (± SE) feeding damage (mm^2^) and proportion (%) of damaged to undamaged seedlings caused by adult *Psylliodes chrysocephala* in a semi‐field experiment. Multipot‐trays containing 10 seedlings of the same accession per tray were arranged in a randomized complete block design with 8 blocks and placed within a *Brassica napus* crop in September for seven days. Different letters indicate significant difference between accessions (*P* < 0.05). Treatment seedlings: *Brassica napus* cv. Arabella, ‘Resynth’ = *B. napus* resynthesized by hybridizing *Brassica rapa* x *Brassica oleracea* using seeds with different seed age (old = 9 years, new = 1 year ), *Sinapis alba* cv. Passion and ‘S. alba2’ breeders line.

### 3.3 Metabolite profiles

A total of ten glucosinolates (GLS) were identified in the seedlings of the *B. napus* lines. The lowest total GLS content was detected in *B. napus* cv. Arabella (13.41 ± 0.78 ng mg^−1^ DW), while the highest was recorded in *S. alba* cv. Passion (269.2 ± 15.71 ng mg^−1^ DW). In all *S. alba* accessions, sinalbin was the only GLS detected (Table 1). In contrast, the aliphatic GLS progoitrin was predominant in the *B. napus* accessions, accounting for 71% of the total GLS content in ‘Arabella’ and 82% in both ‘Resynth (old)’ and ‘Resynth (new)’.

**Table 1:** Glucosinolate content (in mmol μg^−1^ dry weight) found in cotyledons of *Brassica napus* cultivar Arabella, a resynthesized *B. napus* accession (Resynth) grown from seeds of different age (new, old) and three accessions of *Sinapis alba* (Passion, S.alba1, S.alba2)

	Accession											
Glucosinolate	Arabella		Resynth (old)		Resynth (new)		Passion		S.alba1		S.alba2	
	mean	SE	mean	SE	mean	SE	mean	SE	mean	SE	mean	SE
Epiprogoitin	0.18	0.01a	2.97	0.16b	4.60	0.72b	–	–	–	–	–	–
Glucoalyssin	0.27	0.01a	0.46	0.02b	0.45	0.04b	–	–	–	–	–	–
Glucobrassicanapin	0.25	0.02a	3.08	0.06c	5.39	0.38b	–	–	–	–	–	–
Glucobrassicin	1.48	0.10a	2.61	0.05c	2.13	0.14b	–	–	–	–	–	–
Glucoerucin	0.19	0.03ab	0.12	0.01b	0.23	0.01a	–	–	–	–	–	–
Gluconapin	0.53	0.02a	9.06	0.71b	7.76	0.42b	–	–	–	–	–	–
Gluconapoleiferin	0.62	0.03a	1.50	0.08b	1.87	0.14b	–	–	–	–	–	–
Gluconasturtiin	0.26	0.02a	2.42	0.10b	1.81	0.12c	–	–	–	–	–	–
Neoglucobrassicin	0.13	0.00a	0.17	0.01c	0.21	0.01b	–	–	–	–	–	–
Progoitin	9.53	0.52a	98.90	2.91b	111.95	3.24b	–	–	–	–	–	–
Sinalbin	–	–	–	–	–	–	269.20	15.71a	120.98	4.06b	167.19	24.93b
Total	13.41	0.78a	121.3	4.03b	136.4	5.17b	269.20	15.71c	120.98	4.06b	167.19	24.93b

Sample size: n = 5. Different lowercase letters indicate a significant difference between accessions (*P* < 0.05).

Notable variation in GLS composition was observed within the ‘Resynth’ accessions (Table 1): seedlings from ‘Resynth (new)’ exhibited significantly higher levels of glucobrassicanapin, glucoerucin, and neoglucobrassicin compared to ‘Resynth (old)’, whereas seedlings from ‘Resynth’ (old) contained significantly greater amounts of glucobrassicin and gluconasturtiin.

The GC‐MS analyses identified a total of 67 metabolites (Supporting Information, Table S1), revealing significant differences in the central metabolome composition among the accessions (Figure 4). Hierarchical clustering of metabolite profiles reflected species‐level separation between *S. alba* and *B. napus*. Furthermore, the accessions ‘Arabella’ and ‘Resynth’ clustered into separate groups, with the latter further subdividing into ‘old’ and ‘new’. Notably, ‘S.alba2’ and 'Passion' clustered more closely together, while ‘S.alba1’ formed a distinct group (Figure 4). The majority of identified compounds belonged to the central metabolome, with sugars representing the most abundant class. Among these, glucose and fructose exhibited the highest overall concentrations.

**Figure 4:**
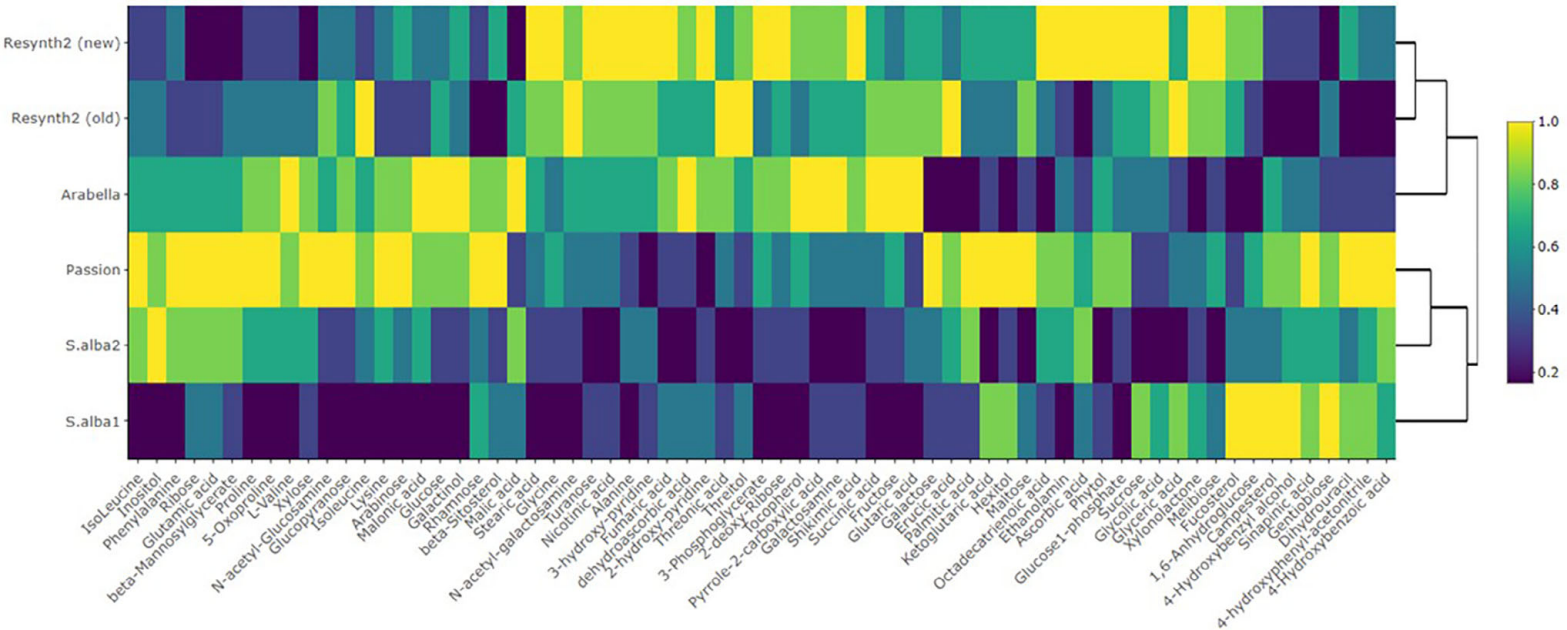
Heatmap of central metabolites of cotyledons of *Brassica napus* cv. Arabella, a resynthesized *B. napus* accession (‘Resynth’) grown from seeds of different age (‘new’ = 1 year, ‘old’ = 9 years) and *Sinapis alba* (‘Passion’, ‘S.alba1’, ‘S.alba2’). The metabolites are hierarchically clustered, n = 6.

All *S. alba* accessions (‘S.alba1’, ‘S.alba2’ and ‘Passion’) exhibited significantly higher levels of specific metabolites, notably 4‐hydrobenzyl alcohol (χ^2^ = 26.68, df = 5, *P* < 0.001), 4‐hydroxybenzoic acid (χ^2^ = 26.31, df = 5, *P* < 0.001), sinapinic acid (χ^2^ = 26.49, df = 5, *P* < 0.001), and gentiobiose (χ^2^ = 23.78, df = 5, *P* < 0.001) compared to the *B. napus* accessions (‘Arabella’, ‘Resynth (old)’, ‘Resynth (new)’) (Figure 5). Conversely, nicotinic acid (χ^2^ = 28.06, df = 5, *P* < 0.001), threonic acid (χ^2^ = 24.55, df = 5, *P* < 0.001), 2‐hydroxy‐pyridine (χ^2^ = 23.32, df = 5, *P* < 0.001), and 3‐hydroxy‐pyridine (χ^2^ = 22.14, df = 5, *P* < 0.001) were found to be present in significantly higher amounts in the *B. napus* accessions. The levels of campesterol were significantly reduced (χ^2^ = 26.91, df = 5, *P* < 0.001) in both ‘Resynth (new)’ and ‘Resynth (old)’ accessions compared to all other accessions (Figure 5). The *B. napus* ‘Resynth (old)’ showed a significantly (χ^2^ = 23.65, df = 5, *P* < 0.001) higher concentration of erucic acid than all other accessions tested.

**Figure 5:**
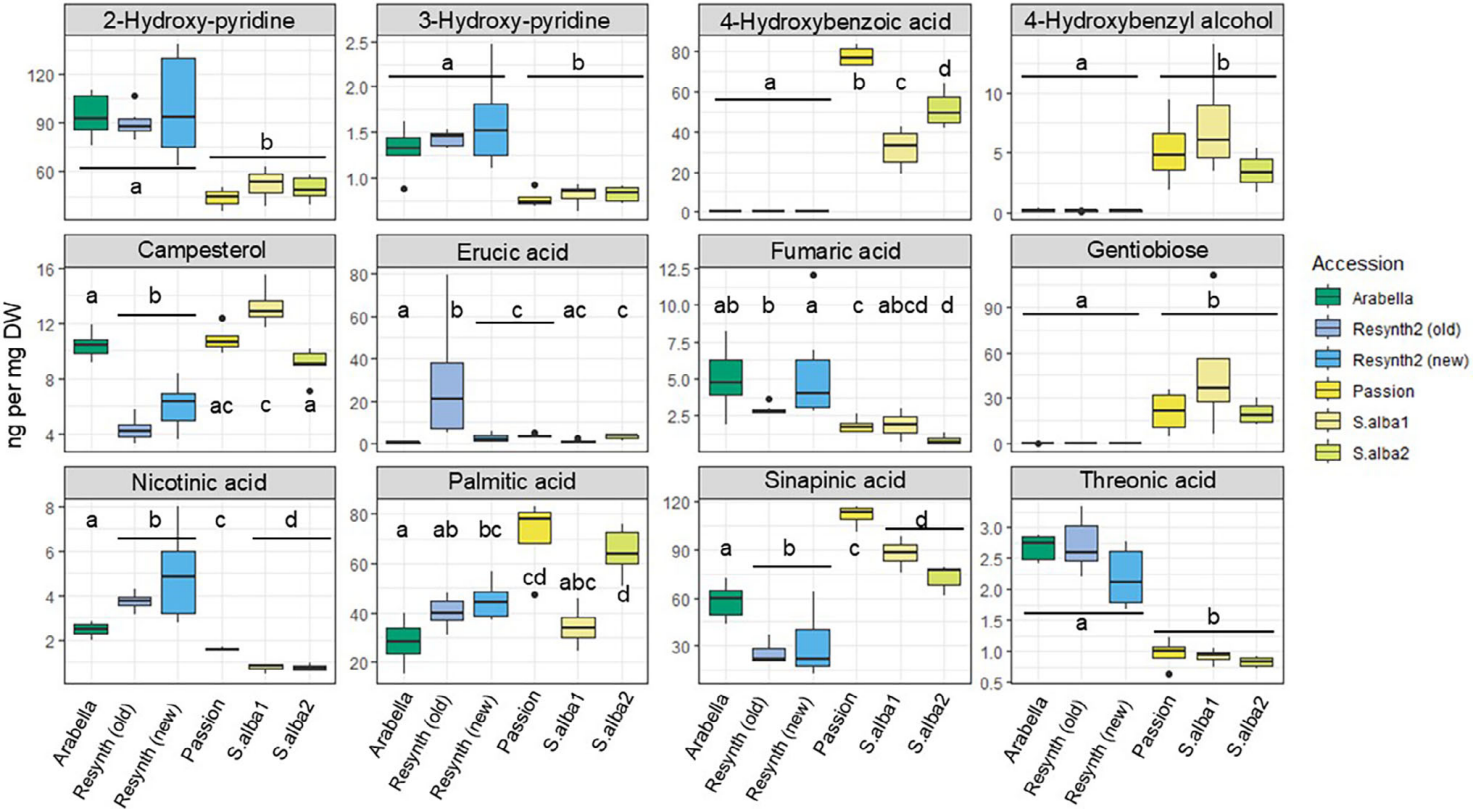
Metabolites with the most contrasting concentrations (ng mg^−1^ dry weight) found in cotyledons of a *Brassica napus* cultivar (‘Arabella’), a resynthesized *B. napus* accession (‘Resynth’) grown from seeds of different age (new, old) and three accessions of *Sinapis alba* (‘Passion’, ‘S.alba1’, ‘S.alba2’). Sample size: n = 6. Different lowercase letters indicate a significant difference between accessions (*P* < 0.05). The median is shown by the central line in each boxplot, with the lower and upper box edges denoting the 25th (Q1) and 75th (Q3) percentiles.

### 3.4 Dual‐choice bioassay on leaf discs

In most cases, the application of metabolites to leaf discs had no significant effect on adult *P. chrysocephala* feeding preference (Figure 6). The strongest effect, as measured by the preference index (PI), was observed for blend‐B (blend‐A plus sinalbin), which caused significant deterrence (PI = ‐0.39, SE = 0.15; *P* = 0.015). Similarly, blend‐A (PI = −0.34, SE = 0.12; *P* = 0.007) and sinapinic acid (PI = −0.32, SE = 0.17; *P* = 0.044) also showed a deterring effect. In contrast, blend‐C, which contained a lower concentration of sinalbin, had no effect on feeding preference (PI = 0.15, SE = 0.14; *P* = 0.335). The highest PI was found for fumaric acid (PI = 0.2, SE = 0.17), however, this effect was not statistically significant (*P* = 0.237).

**Figure 6:**
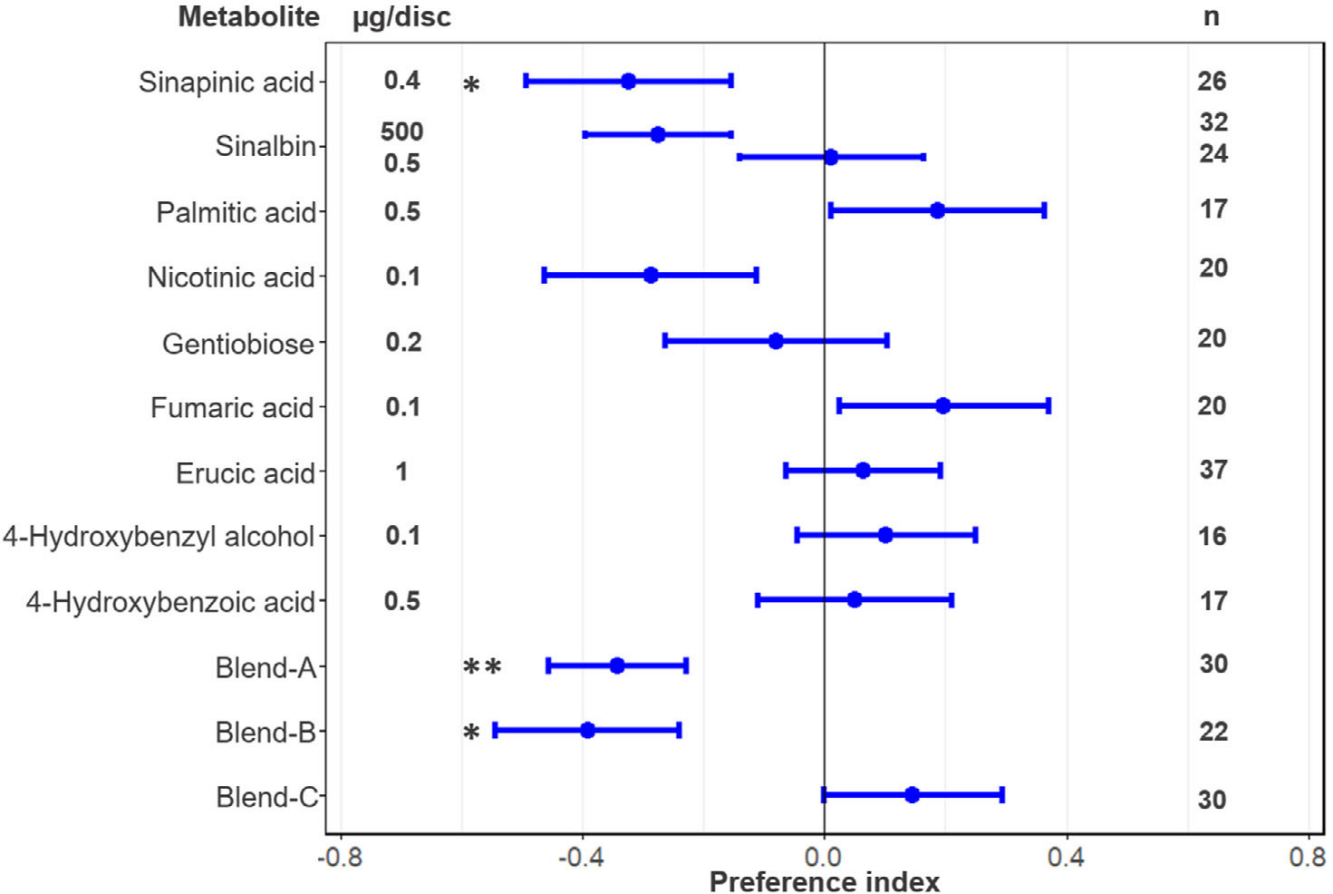
Feeding preference (mean ± SE) of *Psylliodes chrysocephala* adults in dual‐choice bioassays. Respective compounds in solvent were applied on a *Brassica napus* leaf disc and tested against a control disc (only solvent). Blend‐A consisted of 4‐hydroxybenzoic acid, 4‐hydroxybenzyl alcohol, sinapinic acid, gentiobiose and palmitic acid in equal portions of the respective concentrations tested solo. Blend‐B: identical to blend‐A, plus sinalbin (500 μg/leaf disc). Blend‐C: identical to blend‐A plus sinalbin (5 μg/leaf disc). A negative preference index implies a higher feeding on the control disc. Statistical comparison by Wilcoxon one sample test to zero (*: *P* < 0.05; **: *P* < 0.01).

## 4 DISCUSSION

This study identified significant variation in the resistance of Brassicaceae seedlings to feeding by adult *Psylliodes chrysocephala*, confirming both inter‐ and intraspecific variation. Under controlled conditions, we found that the cotyledons of the tested *S. alba* accessions suffered less feeding damage than the standard *B. napus* cv. Arabella. This observation is consistent with findings from various Canadian studies that specifically investigated the resistance of *S. alba* to flea beetle (*Phyllotreta* spp.) feeding.^29^, ^30^, ^31^ Notably, documented resistance was not only found in *S. alba* but also in *S. alba* x *B. napus* hybrids against *Phyllotreta* feeding.^30^ Field experiments in Estonia showed similar numbers of adult flea beetles (*Phyllotreta* ssp.) in *B. napus* and *S. alba* crops, but feeding damage was always lower on cotyledons and true leaves of *S. alba*.^45^ In our semi‐field experiment, the damaged leaf area of *S. alba* seedlings was only half as high as *B. napus* cv. Arabella, but this difference was not significant.

To the best of our knowledge, there are no published data specifically addressing the resistance of *S. alba* seedlings against feeding by adult *P. chrysocephala*. Previous studies investigating feeding on true leaves of more advanced growth stages of *S. alba* found no significant differences in flea beetle feeding damage in both no‐choice tests^46^ and choice tests.^36^ In contrast, our observations demonstrate that *S. alba* seedlings exhibit significantly lower susceptibility to *P. chrysocephala* feeding under controlled conditions. Together, these findings suggest that the susceptibility of *S. alba* to adult *P. chrysocephala* may be influenced by the developmental stage of the plant, with seedlings potentially being less vulnerable compared to more mature plants. A similar ontogenetic effect of *S. alba* was found for the interaction with *P. cruciferae*.^47^ A potential bias in our experiment is that the beetles used in the no‐choice assays were collected from *B. napus* fields and subsequently reared on *B. napus* cv. Arabella before being used in experiments. This prior exposure may have conditioned the beetles to *B. napus* cv. Arabella and could therefore have influenced their feeding on other species and accessions, as shown for *Phaedon cochleariae*.^48^

Metabolome analyses revealed significant variations among the metabolome profiles of the tested accessions. Certain contrasting and characteristic metabolites were further tested in dual‐choice bioassays in which sinapinic acid was the only solitary tested compound to show a significant effect on *P. chrysocephala* feeding preference. Notably, the deterring effect observed for blend‐A (comprising sinapinic acid, 4‐hydroxybenzoic acid, 4‐hydroxybenzyl alcohol, gentiobiose and palmitic acid) and blend‐B (identical to blend‐A plus sinalbin) were comparable in strength to sinapinic acid alone. This similarity suggests that the other components present in blend‐A and ‐B do not contribute interactively to the deterrent effect.

Sinapinic acid, a hydroxycinnamic acid, is widely distributed throughout the plant kingdom, including numerous Brassicaceae species.^49^, ^50^, ^51^ Hydroxycinnamic acids, which belong to the family of phenylpropanoids, are known for conferring traits related to disease resistance, hormone signaling, and cell wall strengthening.^52^ A significant reduction of oviposition by *Spodoptera litura* was found when sinapinic acid was applied to *Capsicum annum* leaves.^53^ Interestingly, feeding by second instar *S. litura* larvae induced elevated sinapinic acid levels in *C. annum*, highlighting its potential role in herbivore resistance. Similarly, an increase of sinapinic acid and gentiobiose in *Brassica nigra* leaves was observed upon herbivory by larval stages of cabbage white butterfly (*Pieris rapae*), along with increased levels of hydroxycinnamic acid derivatives, such as feruloyl‐sinapoyl gentiobiose, and disinapoyl‐gentiobiose.^22^

In the context of our study, these findings are particularly relevant because we detected high amounts of gentiobiose (20–47 ng/mg^−1^ DW) in all tested *S. alba* accessions, whereas only traces (0.05–0.08 ng/mg^−1^ DW) were found in the *B. napus* accessions. The application of gentiobiose itself on leaf discs had no significant effect on feeding preference, but the high levels of gentiobiose and sinapinic acid found in *S. alba* cotyledons could indicate that these compounds might serve as building blocks for hydroxycinnamic acid derivatives like feruloyl‐sinapoyl gentiobiose and disinapoyl‐gentiobiose. A possible contribution of feruloyl‐sinapoyl gentiobiose to the resistance of a *Brassica oleracea* var. *capitata* cultivar against larvae of the lepidopteran *Mamestra brassicae* was found by Rodriguez *et al*. (2023).^23^ When comparing the metabolomes of a resistant cabbage cultivar to a susceptible one, the absence of feruloyl‐sinapoyl gentiobiose in the latter suggests its potential relevance for cabbage in herbivore resistance.

Taken together, our results indicate that the high basal levels of gentiobiose and sinapinic acid observed in *S. alba* cotyledons may reflect a metabolic capacity to form hydroxycinnamic acid conjugates associated with herbivore resistance. This provides a plausible biochemical explanation for the enhanced resistance observed in *S. alba* compared with *B. napus*, even though gentiobiose alone did not directly affect feeding behavior in our assays.

In contrast to our findings, the concentration of hydroxycinnamic acid derivatives was found to be positively correlated with *Brassicogethes aeneus* feeding on flower buds of different brassicaceous species, including *B. napus* and *S. alba*.^42^ While the role of increased sinapinic acid levels in enhancing insect resistance needs further research, it is well documented that high concentrations of sinapinic acid in *Brassica napus* seeds reduce the nutritional value of seed cake, a major byproduct of oilseed processing used in animal feed, due to its anti‐nutritive properties.^54^ This makes elevated sinapinic acid contents undesirable in breeding programs when the compound accumulates in seeds. In contrast, increasing sinapinic acid in leaves, which are not used for feed, does not pose this issue, and may therefore be less of a concern in the context of plant defense.

Sugars, particularly sucrose, are widely recognized as feeding stimulants for insects.^18^, ^55^ Behavioral and electrophysiological responses of *P. chrysocephala* to sugars have been demonstrated by Bartlet et al. (1994)^26^ and Isidoro et al. (1998).^56^ Incorporating fructose, glucose, or sucrose into agar stimulated the feeding of *P. chrysocephala*, with sucrose eliciting the most robust response. Additionally, when the GLS sinigrin was co‐presented with fructose or sucrose, a synergistic effect was observed.^26^ This synergism between host‐specific glucosinolates and sugars as general phagostimulants is also evident in the Brassica‐specialist *Pieris brassicae*.^57^ While Hervé *et al*. (2014)^18^ established a correlation between sucrose content in *B. napus* perianths and *B. aeneus* feeding damage, our experiments did not indicate a direct relationship between sugar content of cotyledons and *P. chrysocephala* feeding. Nevertheless, sugar profiles varied between and within species, which may suggest potential complexities in the interplay between sugar composition and interactions with other metabolites affecting *P. chrysocephala* herbivory.

The differences in total GLS contents among the tested accessions were not associated with the variation in susceptibility towards adult *P. chrysocephala* feeding. The cotyledons of cv. Arabella exhibited only ca. 10% of the total GLS contents of ‘Resynth’ (old and new), but while ‘Resynth (old)’ was consistently less susceptible to feeding, ‘Resynth (new)’ did not significantly suffer more feeding damage than cv. Arabella, making the total GLS content unlikely to be a resistance factor. Similarly, Bartlet *et al*. (1996)^58^ did not find that total GLS levels of different *B. napus* cultivars affected the feeding preference of *P. chrysocephala* adults in multiple‐choice experiments, with GLS levels varying up to five‐fold. On the contrary, Giamoustaris and Mithen (1995)^28^ identified a positive correlation between leaf GLS levels and leaf damage by *P. chrysocephala* on *B. napus*. In a field experiment, the *B. napus* cultivar with a very low seed GLS content was most damaged in the cotyledon stage, although the effect was weak.^27^ Together with our results, these studies suggest that total GLS concentration alone does not consistently predict adult *P. chrysocephala* feeding and that additional factors, such as GLS composition, plant developmental stage, and/or experimental conditions, may modulate beetle responses.

Individual GLS compounds rather than total GLS content may be more relevant for driving interactions with *P. chrysocephala*. Progoitrin was the most abundant GLS in all tested *B. napus* accessions in the present study. Previous work has shown that progoitrin content can positively correlate with *P. chrysocephala* larval performance, as reflected by increased larval weight, whereas no such relationship was observed for total GLS content.^35^ This suggests that elevated progoitrin levels may enhance host suitability for larvae, even if they do not increase susceptibility to adult feeding. Despite resynthesized accessions ‘Resynth’ (old and new) exhibiting approximately ten‐fold higher progoitrin levels than cv. Arabella, lower feeding damage was repeatedly found on the cotyledons of ‘Resynth’ (old). This indicates that progoitrin does not act as a deterrent to adults and may exert different effects across life stages.

Sinalbin is the predominant GLS found in seeds of *S. alba*,^59^, ^60^ and it was the only GLS identified in the cotyledons of our *S. alba* samples. Similarly, Hopkins *et al*. (1998)^61^ found sinalbin to comprise more than 90% of the glucosinolate content in the cotyledons of *S. alba* seedlings. The *S. alba* cultivar ‘Passion’ contained ca. twice as much sinalbin compared to the other *S. alba* accessions, but no significant difference in feeding damage occurred between them. In the choice tests, applying sinalbin to *B. napus* leaf discs at the physiological concentration found in *S. alba* cotyledons did not lead to a significant response, but there was a tendency for an antifeedant effect. Also, the deterrent effects of blend‐A and blend‐B were on the same level, indicating that the addition of the physiological concentration of sinalbin found in *S. alba* to blend‐B does not alter the feeding preference. Interestingly, the deterrent effect found for blends‐A and ‐B were neutralized when only a small amount of sinalbin was present in the blend (blend‐C). Similar findings were obtained by Bodnaryk (1991),^47^ who found high sinalbin concentrations in seedlings of *S. alba* had an antixenotic effect on adult *P. cruciferae* feeding, but when reduced concentrations were presented, feeding was increased. These findings suggest a dose‐dependent effect with contrasting responses, in which low concentrations of sinalbin appear to act as a phagostimulant for the Brassica specialists *P. chrysocephala* and *P. cruciferae*, whereas higher concentrations have antixenotic effects.

In the no‐choice bioassays, higher feeding activity was observed when using pre‐aestivated than post‐aestivated beetles. Similar findings were reported by Ankersmit (1964),^5^ who measured daily feeding rates. Adult *P. chrysocephala* undergo obligatory aestivation and the period between adult emergence and the onset of aestivation is short, compared to the post‐aestivation period.^5^, ^6^ The increased feeding during pre‐aestivation is likely linked to the substantial metabolic shifts observed in this phase, as beetles accumulate lipid reserves and adjust energy metabolism prior to entering aestivation.^6^

Our investigation revealed a notable influence of seed age on seedling susceptibility to *P. chrysocephala* feeding and on the accumulation of several metabolites. Significant differences were observed in fumaric acid, erucic acid, and glucosinolates (e.g., glucobrassicanapin, glucoerucin, gluconasturtiin) between ‘Resynth (old)’ and ‘Resynth (new)’. These age‐related shifts in metabolite levels may underlie the observed variation in susceptibility to *P. chrysocephala* feeding. Among the metabolites, erucic acid showed the most pronounced change, being markedly elevated in cotyledons from older seeds. Although erucic acid is traditionally undesirable in *B. napus* breeding due to negative implications for seed quality, its unique physicochemical properties also make it attractive for industrial applications, including lubricants, plasticizers, and other specialty chemicals.^62^ Soroka and Grenkow (2013)^31^ proposed that a reduced erucic acid content in *S. alba* may enhance vulnerability to insect herbivory, which is consistent with the high erucic acid levels of ‘Resynth (old)’ and its comparatively lower susceptibility. Although our dual‐choice assays did not demonstrate a direct influence of erucic acid on *P. chrysocephala* feeding, the potential role of erucic acid in mediating plant‐insect interactions warrants further investigation.

It should be noted that the no‐choice bioassays and metabolite analyses were conducted on cotyledons, whereas the leaf disc choice assays were performed using true leaves from BBCH14 plants. This difference in plant tissue and developmental stage may substantially influence phagostimulant or repellent properties, as both chemical composition and physical characteristics change during plant development. Such ontogenetic and tissue‐specific differences in the feeding substrate could potentially mask or modify the effects of individual compounds detected in the metabolite analysis, thereby contributing to the lack of a direct correspondence between metabolite profiles and feeding responses observed in the bioassays.

To our knowledge, insect resistance linked specifically to seed age has not been previously reported. Future research could investigate how ageing‐induced shifts in metabolite profiles translate into altered resistance phenotypes. It would also be valuable to assess the role of epicuticular wax composition and structure, which was not examined in the present study. Epicuticular waxes have been shown to influence feeding and host selection in other Brassicaceae herbivores, suggesting they could contribute to the observed differences in susceptibility.^63^, ^64^ Such insights may inform breeding strategies to target certain metabolites, while ensuring that commercially distributed seed lots continue to rely on non‐aged, high‐vigor seeds.

## 5 CONCLUSION

This study demonstrates that *S. alba* seedlings show significantly greater resistance to *P. chrysocephala* feeding than *B. napus* under controlled conditions, highlighting notable interspecific variation. Metabolomic analyses and feeding bioassays identified sinapinic acid as a key deterrent, but small amounts of sinalbin can reverse this effect.

Another novel finding is the strong influence of seed age: seedlings germinated from older seeds experienced less feeding damage and showed altered metabolite profiles, including elevated erucic acid than those grown from young seeds. While the precise mechanisms remain unclear, these results suggest that seed physiology and associated metabolic shifts can impact seedling resistance to herbivores.

More field‐realistic field studies are needed to support our findings. However, this study can help develop *B. napus* cultivars resistant to adult *P. chrysocephala*, thereby supporting integrated pest management programs and offering growers strategies to reduce pest damage through seed selection.

## Supporting information


**Figure S1.** Setup of the no‐choice bioassays without lid.
**Table S1**. Metabolites (in ng/mg DW) found in cotyledons of *Brassica napus* cv Arabella, a resynthesized *B. napus* accession (‘Resynth’) grown from seeds of different ages (new = 1 year, old = 9 years) and three accessions of *Sinapis alba* (‘S.alba1’, ‘S.alba2’, ‘Passion’). Different lowercase letters indicate a significant difference between accessions (*P* < 0.05).

## Data Availability

The data that support the findings of this study are available from the corresponding author upon reasonable request.
