# Supplementary material for: Seedling resistance and chemical defenses against Psylliodes chrysocephala : the roles of seed age and sinapinic acid in Sinapis alba and Brassica napus
Source: Pest Manag Sci. 2026 Mar 18;82(11):10727–38. doi: 10.1002/ps.70724 (PMC13665650; doi:10.1002/ps.70724)
Supplement: Supplementary file 1 [file PS-82-10727-s001.docx]

**Supplementary Material: Seedling resistance and chemical defenses against *Psylliodes chrysocephala*: the roles of seed age and sinapinic acid in *Sinapis alba* and *Brassica napus***

Rüde, D., Cook, S.M., Ulber, B., Pöhnl, T., & Rostás, M.

**Figure S1**: Setup of the no-choice bioassays without lid.

**Table S1:** Metabolites (in ng/mg DW) found in cotyledons of *Brassica napus* cv Arabella, a resynthesized *B. napus* accession (‘Resynth’) grown from seeds of different ages (new = 1 year, old = 9 years) and three accessions of *Sinapis alba* (‘S.alba1’, ‘S.alba2’, ‘Passion’). Different lowercase letters indicate a significant difference between accessions (P < 0.05).
